# Microbiome-Transcriptome Interactions Related to Severity of Respiratory Syncytial Virus Infection

**DOI:** 10.1038/s41598-019-50217-w

**Published:** 2019-09-25

**Authors:** Abhijeet R. Sonawane, Liang Tian, Chin-Yi Chu, Xing Qiu, Lu Wang, Jeanne Holden-Wiltse, Alex Grier, Steven R. Gill, Mary T. Caserta, Ann R. Falsey, David J. Topham, Edward E. Walsh, Thomas J. Mariani, Scott T. Weiss, Edwin K. Silverman, Kimberly Glass, Yang-Yu Liu

**Affiliations:** 1 Channing Division of Network Medicine, Brigham and Women’s Hospital and Harvard Medical School, Boston, MA 02115 USA; 2 Department of Physics, Hong Kong Baptist University, Kowloon Tong, Hong Kong SAR, China; 3 Departments of Pediatrics, University of Rochester Medical Center, Rochester, NY 14642 USA; 4 Department of Biostatistics and Computational Biology, University of Rochester Medical Center, Rochester, NY 14642 USA; 5 Department of Microbiology and Immunology, University of Rochester Medical Center, Rochester, NY 14642 USA; 6 Department of Medicine, University of Rochester Medical Center, Rochester, NY 14642 USA; 7 Center for Cancer Systems Biology, Dana Farber Cancer Institute, Boston, MA 02115 USA; 8 State Key Laboratory of Environmental and Biological Analysis, Hong Kong Baptist University, Hong Kong, SAR China

**Keywords:** Gene expression analysis, Data integration, Microbiology, Reverse engineering, Infectious diseases

## Abstract

Respiratory syncytial virus (RSV) is a major cause of lower respiratory tract infections and hospital visits during infancy and childhood. Although risk factors for RSV infection have been identified, the role of microbial species in the respiratory tract is only partially known. We aimed to understand the impact of interactions between the nasal microbiome and host transcriptome on the severity and clinical outcomes of RSV infection. We used 16 S rRNA sequencing to characterize the nasal microbiome of infants with RSV infection. We used RNA sequencing to interrogate the transcriptome of CD4^+^ T cells obtained from the same set of infants. After dimension reduction through principal component (PC) analysis, we performed an integrative analysis to identify significant co-variation between microbial clade and gene expression PCs. We then employed LIONESS (Linear Interpolation to Obtain Network Estimates for Single Samples) to estimate the clade-gene association patterns for each infant. Our network-based integrative analysis identified several clade-gene associations significantly related to the severity of RSV infection. The microbial taxa with the highest loadings in the implicated clade PCs included *Moraxella*, *Corynebacterium*, *Streptococcus, Haemophilus influenzae*, and *Staphylococcus*. Interestingly, many of the genes with the highest loadings in the implicated gene PCs are encoded in mitochondrial DNA, while others are involved in the host immune response. This study on microbiome-transcriptome interactions provides insights into how the host immune system mounts a response against RSV and specific infectious agents in nasal microbiota.

## Introduction

Respiratory syncytial virus (RSV) is a major cause of lower respiratory tract infections and hospital visits during infancy and childhood^1–3^. Almost all children will have been infected with RSV by age two, with about 3% requiring hospitalization. A recent report estimated that in 2015 about 33.1 million children worldwide under age 5 had RSV-related acute lower respiratory infection; of those, approximately 10% required hospitalization with 59,000 in-hospital fatalities^1^. The incidence and mortality of RSV vary between geographic locations, with children from developing countries considered to be at higher risk^4^. In the United States, 20% of infants require outpatient treatment for RSV-related illnesses, with an associated economic burden of $1.9 billion^5^; around 3% of these cases are associated with serious bronchiolitis and viral pneumonia–the most common forms of severe RSV infection. In addition to major risk factors such as congenital or chronic cardiopulmonary disease, meta-analyses have identified additional risk factors for RSV, including preterm birth, low birth weight, siblings at home, day-care attendance, and maternal smoking^6–8^. Infants with RSV infection in early childhood are also at a higher risk of developing asthma and wheezing^9–11^. Although prophylactic treatments are available to prevent RSV infection in the most at-risk infants, attempts are ongoing to identify an effective and safe vaccine or small molecule drug to reduce the health burden of RSV.

Biomarker discovery through expression analysis is an important step in assessing disease severity and in distinguishing RSV from other common respiratory viruses^12–14^. Transcriptional profiles often reflect a host’s immune response to the virus, helping to explain disease progression and characterize its severity. Several studies have reported that T cell mediated response is crucial in clearing the viral load during RSV infection^15,16^. Recent reports also indicate that RSV infects CD4^+^ and CD8^+^ T cells and affects T cell function^12,17,18^, implicating T cells as potential biomarkers for RSV severity.

The microbiome in the respiratory tract is known to influence the course of acute infectious diseases^19^. The succession pattern of the nasal microbial community might influence host responses to RSV, thereby modulating inflammation and possibly disease severity. Indeed, several studies indicate that the nasal microbial composition affects the overall risk of developing respiratory tract infections^20,21^ and is associated with the severity of acute respiratory symptoms^22^.

Joint characterization of the nasal microbiome and host transcriptome may provide valuable insights into how viral infections influence the host response. However, the impact of the interactions between the nasal microbiome and the host transcriptome on the severity and clinical outcomes of RSV infection has not been fully understood. Recent studies have reported associations between nasal microbial compositions and whole blood gene expression^22^. In this work, we perform an integrative analysis to study associations between nasal microbial compositions and transcriptional profiles of bloodstream CD4^+^ T cells.

**Figure 1:**
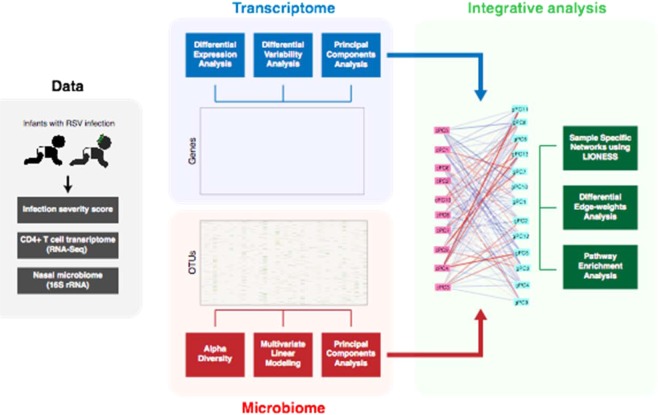
An overview of the data and analyses performed in this study.

Network approaches provide an important framework for understanding complex relationships that influence human health^23^. In our study, we construct a network model that correlates microbial taxonomic profiles and host transcriptomic profiles. Examining this network in the context of RSV severity highlights important patterns of transcriptomic response related to immune processes and viral infection. An overview of our analysis approach is summarized in Fig. 1.

## Results

### Baseline characteristics of participants and data source

**Figure 2:**
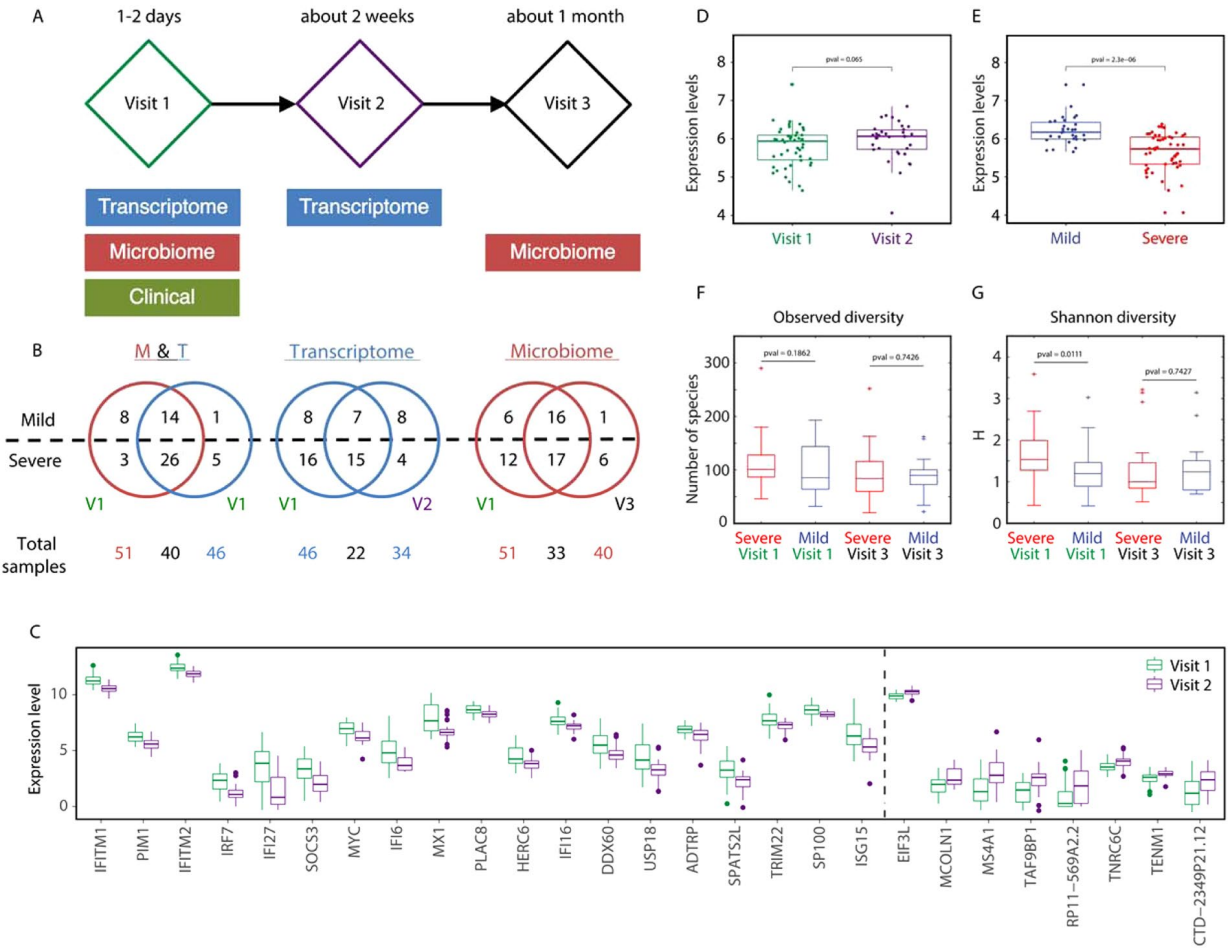
(**A**) Overview of study timelines, including when each type of data was collected. All 58 infants included in this study had both transcriptomic and microbiome data collected for at least one time point. (**B**) Venn diagrams showing the overlap in the infants that had either transcriptomic or microbiome data collected during the acute visit (57 of the 58 infants), the overlap in the transcriptomic data collected at either the initial (Visit 1) or follow-up visit (Visit 3), and the overlap in the infants microbiome data collected at either the initial (Visit 1) or follow-up visit (Visit 2). Numbers are shown separately for mild and severe groups, with the total number in each category indicated below the Venn diagram. (**C**) Differential gene expression analysis comparing Visit 1 versus Visit 2 RNA-Seq samples yields 27 significantly differentially expressed genes (FDR < 0.05). The log2 of the expression levels of these genes are shown as boxplots combining mild and severe samples. The genes on the left side of the panel have higher mean expression during the acute visit and those on the right side of the panel have higher expression levels in the follow-up visit (**D**,**E**) The log2 expression levels for EZH1 comparing (**D**) data from infants with severe versus mild RSV infection and (**E**) data from the acute and follow-up visit. The reported FDR *p*-values are based on *limma* analysis. (**F**,**G**) Abundance profiles of nasal microbiota comparing groups of mild and severe infants at each of the two visits, including (**F**) observed diversity and (**G**) Shannon diversity index.

We analyzed data for 58 infants with RSV infection, each of whom had both CD4 + gene expression and nasal microbiome data. The RSV disease severity was measured by the Global Respiratory Severity Score (GRSS). This allowed us to divide infants into two groups based on severity: mild and severe (see Methods section). We emphasize that GRSS is a composite score that reflects the worst values during the entire illness, rather than a single time point, allowing us to address the problem of clinical variability among subjects. GRSS also recapitulates more frequently used factors such as viral load (which has been associated with RSV disease severity in several studies^24,25^) and need for hospitalization (Supplemental Fig. 1), thus acting as excellent surrogate for disease severity^26^. Among the infants, 23 had mild illness and 35 had severe illness. There was no difference in any demographic characteristics between mildly and severely ill subjects (Table 1). The data collection occurred in three stages (Fig. 2A).

**Table 1:** Demographic and clinical information for the 58 infants included in our analysis.

	RSV Severity Group		P Value
	Mild n (%)	Severe n (%)	
**Sex**			
Female	13 (44.83%)	16 (55.17%)	0.592
Male	10 (34.48%)	19 (65.52%)	
**Ethnicity**			
Hispanic or Latino	6 (60%)	4 (40%)	0.173
Non-Hispanic or Non-Latino	17 (35.42%)	31 (64.58%)	
**Race**			
White	14 (36.84%)	24 (63.16%)	0.789
Black or African American	5 (45.45%)	6 (54.55%)	
Other	4 (44.44%)	5 (55.56%)	
**Hospitalization**			
Not Hospitalized	23	0	
Hospitalized (>24 h)	1	34	
	**Mild Mean (SE)**	**Severe Mean (SE)**	P Value
Severity score	1.32 (0.20)	6.37 (0.29)	<0.001
Age at Visit 1	3.81 (0.45)	2.98 (0.37)	0.155
Days since onset relative to Visit 1	4.87 (0.50)	5.21 (0.38)	0.595

### Transcriptome data analysis

We first characterized the gene expression data for these infants, as measured by RNA-sequencing (RNA-Seq) of CD4^+^ T cells collected at two distinct time points, with 46 samples collected during the acute visit (Visit 1) and 34 samples collected at follow-up day 12–16 (Visit 2). Out of 22 subjects who had RNA-Seq samples collected at both the acute and follow-up visit, 7 were mild and 15 were severe (Fig. 2B).

We used principal component analysis (PCA) to investigate the data structure in light of various measured clinical variables (Supplemental Fig. 2). This analysis indicated that enrollment season was a significant contributor to structure in our expression data. Therefore, we applied batch-correction to remove this signal from the data. We then performed differential expression analysis, comparing the expression levels of genes between the acute and follow-up visit. We identified 27 genes that were differentially expressed at a Benjamini-Hotchberg false discovery rate (FDR) less than 0.05 (Fig. 2C). Many of the genes that had higher expression levels in the acute visit are known to be important in mediating host immune response. This included IFITM1 and IFITM2, which are known to inhibit the infection and replication of respiratory syncytial virus^27,28^, IFI27, a known biomarker for RSV^29^, and IRF7, a gene associated with suppression of innate immunity response^30^. Other genes with known associations to RSV and higher expression in the acute visit included SOCS3^31^, MX1^32^, and the ISGylation pathway genes USP18 and ISG15^33^. Our analysis also identified several genes with higher expression in the follow-up visit, including MS4A1, which encodes the CD20 protein, and MCOLN1. CD20 + B cells are prominent in the lung tissue of infants with fatal RSV infection^34,35^ and MS4A1 is upregulated in infants after the administration of live attenuated influenza vaccine, indicating an association with immune system processes^36^. RSV activates innate immunity through the toll like receptor (TLR) pathway. MCOLN1 has been associated with TLR signaling through modulating viral pathogen-associated molecular pattern (PAMP) along with trafficking of single-stranded RNA (ssRNA) into lysosomes^37,38^. MCOLN1 also regulates autophagy^39^, as expected in the convalescence phase of an infection.

We also evaluated if any genes changed their expression levels in response to RSV infection severity. We found 600 genes differentially expressed at a nominal *p*-value significance of less than 0.05. Out of these,  the most significantly differentially-expressed gene was EZH1 (*p*-value = 2.3 × 10^−6^; FDR = 0.026) (Fig. 2D,E). EZH1 was previously included in a biosignature proposed as a molecular diagnosis tool for RSV infection^40^. Interestingly, both EZH1 and IFITM have been implicated in immune response signaling indicating resolving infection^27^.

Because we were only able to identify a handful of genes, primarily related to immune response, as significantly differentially-expressed in these same infants, we also investigated whether differences between severe and mild RSV infection could be captured using other methods. In particular, we examined whether gene expression differences between mild and severe RSV infection might be better captured through changes in variability rather than in mean expression levels. We evaluated the differential variability in gene expression levels between mild versus severe samples using the F-test and identified 641 (5.5%) genes that were differentially variable at an FDR significance threshold of 0.05. Gene Ontology enrichment analysis^41^ identified a number of biological processes nominally associated with these genes (Supplemental Fig. 3). Many of these were associated with mitochondrial activity such as mitochondrial gene expression and translation. Mitochondria are important for viral suppression of the innate immunity^30^.

### Microbiome data analysis

We next characterized microbiome data from nasal swabs obtained by 16 S rRNA sequencing. This included data collected at two time points, with 51 samples collected during the acute visit (Visit 1) and 40 collected during a one-month follow-up visit (Visit 3); 23 of these samples were from infants with mild RSV infection and 35 were from infants with severe RSV infection (Fig. 2B).

We used both PCA and Principal Coordinate Analysis (PCoA) with various dissimilarity metrics, including rooted-Jensen–Shannon divergence (rJSD)^42^ as well as weighted and unweighted Unifrac distance^43^, to reduce dimensionality and visualize the microbiome data (Supplemental Fig. 4)^44^. We also applied MaAsLin (Multivariate association Analysis with Linear modeling) to test for significant relationships between microbial taxa and clinical outcome, after adjusting for sex, race, and enrollment season^45^. At a nominal *p*-value of 0.05, this analysis identified one operational taxonomic unit (OTU) – *H. influenza* – as positively associated with severity, and two OTUs – *Ralstonia* and *Streptococcus* – as negatively associated with severity. We also assessed the influence of visit and infection severity on the microbiome composition by using two measures to quantify the α-diversity: (1) observed number of operational taxonomic units (OTUs), i.e., the OTU richness and (2) the Shannon index^46^. We found that both α-diversity measures have higher values for infants with severe infection compared to those with mild infection during the acute visit (Visit 1), as well as higher values in the acute as compared to the post-acute visit (Visit 3) for infants with severe infection (Fig. 2F,G). In particular, the Shannon diversity is significantly different between severe versus mild samples in Visit 1 (*p*-value = 0.011), and between the Visit 1 and Visit 3 samples among infants with severe RSV infection (*p*-value = 0.0057). The infection score was estimated during Visit 1 (the acute phase). Severity of infection generally decreases over time, potentially explaining why we observe that the α-diversity decreases between the Visit 1 and Visit 3 samples.

### Integrative analysis of transcriptome and microbiome data

Although it has been suggested that host gene expression is influenced by the microbiome, the biological mechanisms that may facilitate these types of interactions are largely unknown^47^. Integrative analysis of microbiome and transcriptome data could help us understand the relationships between host gene expression, microbial composition, and disease pathogenesis. Therefore, we applied a network-based approach, which systematically evaluates the interdependence of multiple biological entities instead of looking at each one independently, to identify microbiome—transcriptomic relationships important for RSV severity.

**Figure 3:**
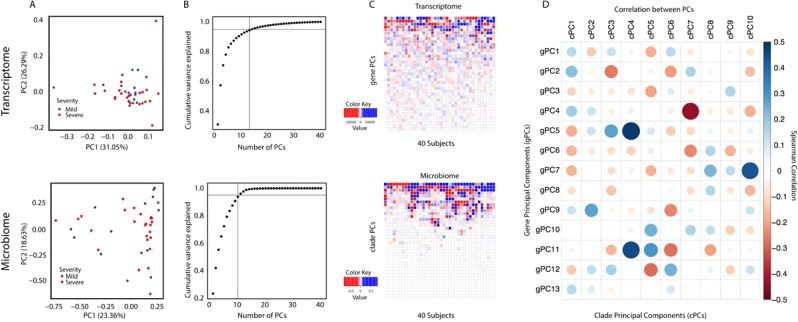
(**A**) Principal component analysis was performed on Visit 1 samples for which we had both transcriptome and microbiome data to reduce the transcriptome into gPCs (top) and the microbiome into cPCs (bottom). (**B**) Cumulative distribution of the amount of variance explained by the top gPCs and cPCs. The vertical and horizontal lines indicate the number of PCs which explain 95% of variance and which were included in our network analysis. (**C**) The coordinates of the gene and clade principal components across the 40 analyzed samples from Visit 1. (**D**) Spearman correlation across the loadings of the top 13 gPCs and the top 10 cPCs.

We focused on the 40 infants from Visit 1 that had paired transcriptome and microbiome data (see Fig. 2B) and performed dimension reduction on these data using PCA (Fig. 3A). This analysis identified 13 gene principal components (gPCs) and 10 clade principal components (cPCs) that explained 95% of the variance in each of their respective data (Fig. 3B).

The loadings of each cPC and gPC represent a pattern of highly correlated microbial and transcriptional abundances, respectively (Fig. 3C). In order to relate these patterns and identify associations between highly varying genes and clades, we calculated the Spearman correlation between the loadings of the top gPCs and cPCs (Fig. 3D). We find several gPC—cPC pairs that are highly correlated, such as gPC5 and cPC4 (most positively correlated; ρ = 0.48), and gPC4 and cPC7 (most negatively correlated; ρ = −0.43). Interestingly, these relationships are not limited to the top few PCs, which are normally the primary focus of dimension reduction analysis. Instead, these relationships highlight that prominent patterns in microbiome data may be associated with more subtle patterns in gene expression, and vice versa.

### Integration of microbiome/transcriptome relationships with clinical characteristics

To interpret these results, we considered this correlation matrix as edges in a bipartite graph, where nodes are gPCs and cPCs. This network framework can help us identify important transcriptomic-microbiomic (gPC—cPC) relationships. However, since this network was derived using information from all samples, by itself it is unable to shed light on which of these relationships might be associated with differences in the various phenotypic or clinical properties of the input samples, including disease severity.

To overcome this limitation, we applied LIONESS^48,49^, which employs a jackknife approach to reverse engineer a set of sample-specific networks, to our gPC/cPC correlation network. This allowed us to construct separate correlation networks between gPCs and cPCs for each of the infants and to analyze gPC—cPC relationships in light of clinical information for these infants.

**Figure 4:**
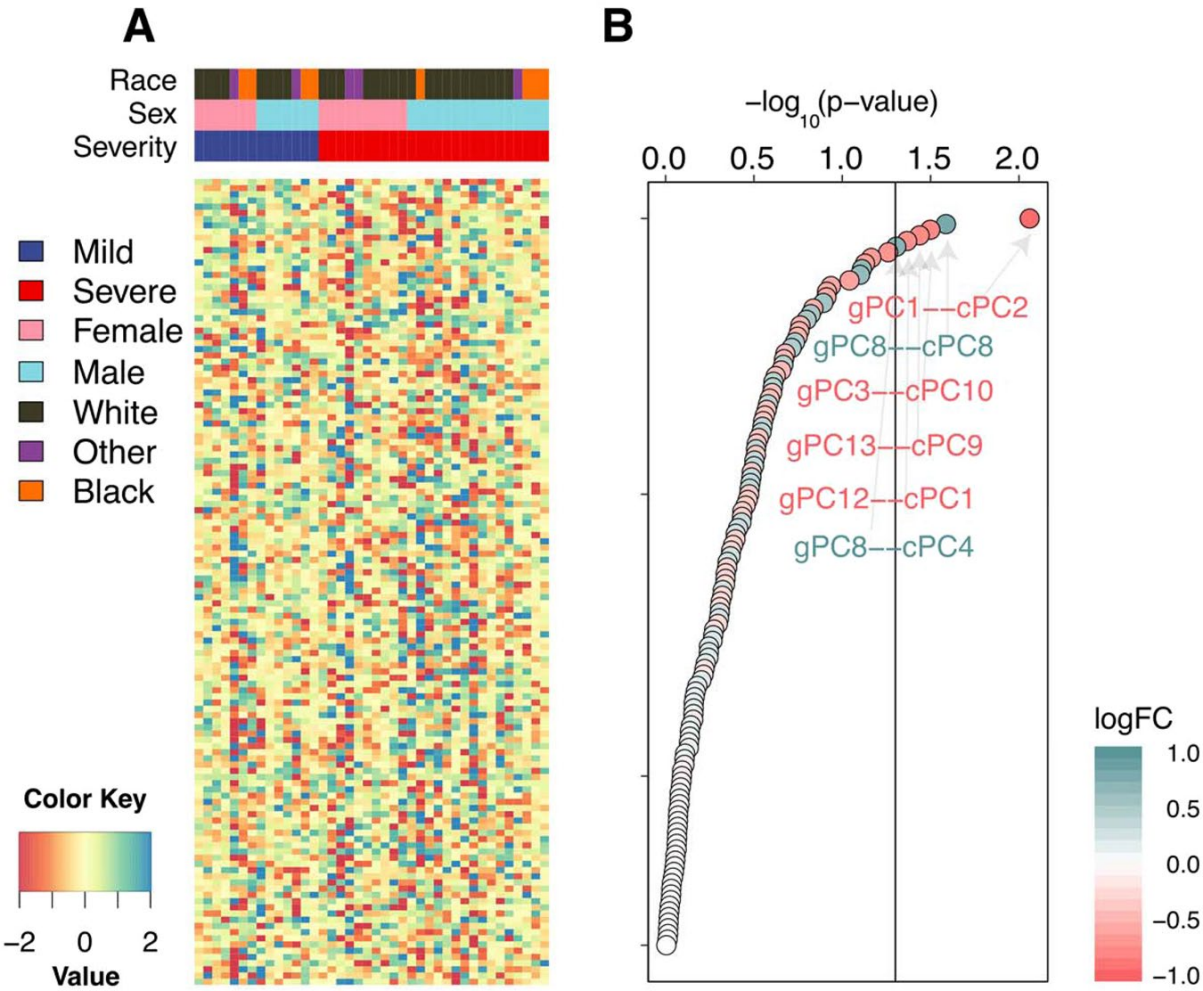
(**A**) The weights of edges predicted for each of the 40 sample-specific networks obtained from the LIONESS analysis. Columns are grouped based on each infant’s infection severity, sex, and race. Rows are sorted based on the significance of edges. (**B**) The significance of each edge as defined from multivariate linear analysis comparing edge-weights between two groups, mild and severe, and corrected for sex and race. The top significant edges with nominal *p*-value less than 0.05 are labeled.

The weights of edges across these networks are shown in Fig. 4A. We compared the distribution of the sample-specific edge-weights between the mild and severe groups and identified six edges which were nominally significant with a *p*-value less than 0.05: gPC1 with cPC2, gPC8 with cPC8 and cPC4, gPC3 with cPC10, gPC13 with cPC9, and gPC12 with cPC1 (Fig. 4B).

To assess the robustness of this result, we performed a sensitivity analysis by randomly selecting 10 samples each from mild and severe groups and repeating this analysis 1000 times. This analysis allows us to assess the robustness of our results given our relatively low sample number as well as the disparity between the number of mild and severe samples. If an edge is robustly different between the severe and mild groups, we will recover that association repeatedly across the randomizations. We found that the top edges obtained above were frequently (at least 20% of the time compared to 5% expected by chance) identified as significant with the same direction of effect (Supplemental Fig. 5).

### Microbiome/transcriptome relationships identify clades that may impact the host immune response

Six clade principal components, cPC2, cPC8, cPC10, cPC9, cPC1, and cPC4 (ordered by significance) were identified as differentially-associated with gPCs in the context of RSV severity based on our network analysis. To understand why these cPCs might have a different relationship with the host transcriptome in the context of RSV severity, we identified their associated top OTUs (Table 2). We find that genus like *Streptococcus, Corynebacterium, Alloiococcus, Haemophilus influenzae*, and *Staphylococcus* are among the top OTUs in cPC2, *Ralstonia*, *Corynebacterium*, *Neisseriaceae* (family, genus not known), and *Pseudomonas* are among the top OTUs in cPC8, and *Corynebacterium, Ralstonia*, *Alloiococcus*, and *Staphylococcus* are among the top OTUs in cPC10. Some of these same OTUs were also identified as OTUs that discriminate between severe and mild RSV infection in our MaAsLin analysis.

**Table 2:** Microbial composition and loadings of the top clades in each of the cPCs identified as associated with significant edges (with gPCs in parentheses) in the sample-specific network analysis. X = unclassified.

Family		Genus	Species	OTU ID	PC loading
**cPC2 (gPC1)**	Streptococcaceae	*Streptococcus*	*X*	4479601	−0.834726
	Corynebacteriaceae	*Corynebacterium*	*X*	4474764	0.378128
	Aerococcaceae	*Alloiococcus*	*X*	886735	0.333977
	Staphylococcaceae	*Staphylococcus*	*X*	1060737	0.147457
	Neisseriaceae	*X*	*X*	861327	−0.074763
	Moraxellaceae	*X*	*X*	932707	0.071822
	Pasteurellaceae	*Haemophilus*	*influenzae*	3125352	0.067097
	Moraxellaceae	*X*	*X*	893147	0.064341
	Moraxellaceae	*Moraxella*	*X*	1080004	0.044229
	Streptococcaceae	*Streptococcus*	*X*	131778	−0.038727
**cPC8 (gPC8)**	Oxalobacteraceae	*Ralstonia*	*X*	550644	0.4919892
	Corynebacteriaceae	*Corynebacterium*	*X*	4474764	−0. 4735642
	Neisseriaceae	*X*	*X*	861327	−0. 4097664
	Corynebacteriaceae	*Corynebacterium*	*X*	997086	0.3448678
	Aerococcaceae	*Alloiococcus*	*X*	886735	0. 2542127
	Streptococcaceae	*Streptococcus*	*Agalactiae*	1076969	−0.2107604
	Moraxellaceae	*X*	*X*	932707	−0.2008934
	Pseudomonadaceae	*Pseudomonas*	*X*	350105	0.1744260
	Streptococcaceae	*Streptococcus*	*X*	4479601	− 0.1421117
	Staphylococcaceae	*Staphylococcus*	*X*	1060737	−0.1144590
**cPC10 (gPC3)**	Corynebacteriaceae	*Corynebacterium*	*X*	997086	0.81347
	Oxalobacteraceae	*Ralstonia*	*X*	550644	−0.460545
	Aerococcaceae	*Alloiococcus*	*X*	886735	−0.208054
	Staphylococcaceae	*Staphylococcus*	*X*	1060737	−0.175109
	Corynebacteriaceae	*Corynebacterium*	*X*	4474764	0.152976
	Neisseriaceae	*X*	*X*	861327	−0.086017
	Moraxellaceae	*X*	*X*	893147	−0.062479
	Streptococcaceae	*Streptococcus*	*X*	4455633	−0.058702
	Corynebacteriaceae	*Corynebacterium*	*X*	484315	0.053777
	Pseudomonadaceae	*Pseudomonas*	*X*	350105	0.050156
**cPC9 (gPC13)**	Aerococcaceae	*Alloiococcus*	*X*	886735	−0.56923761
	Oxalobacteraceae	*Ralstonia*	*X*	550644	0.55397649
	Corynebacteriaceae	*Corynebacterium*	*X*	4474764	0.52806963
	Neisseriaceae	*X*	*X*	861327	−0.18306144
	Staphylococcaceae	*Staphylococcus*	*X*	1060737	−0.15290246
	Moraxellaceae	*X*	*X*	893147	−0.13401397
	Streptococcaceae	*Streptococcus*	*X*	4455633	−0. 08041118
	Pseudomonadaceae	*Pseudomonas*	*X*	350105	0.04756214
	Pasteurellaceae	*Haemophilus*	*influenzae*	3125352	0.04140956
	Streptococcaceae	*Streptococcus*	*X*	4479601	−0.02649437
**cPC1 (gPC12)**	Moraxellaceae	*Moraxella*	*X*	1080004	−0.95754551
	Staphylococcaceae	*Staphylococcus*	*X*	1060737-	0.15074314
	Corynebacteriaceae	*Corynebacterium*	*X*	4474764	0.11808617
	Neisseriaceae	*X*	*X*	861327	0.08584030
	Aerococcaceae	*Alloiococcus*	*X*	886735	0.08026760
	Moraxellaceae	*Moraxella*	*X*	1053321	−0.07771730
	Pasteurellaceae	*Haemophilus*	*influenzae*	3125352	0.07023736
	Streptococcaceae	*Streptococcus*	*X*	4479601	0.06943155
	Moraxellaceae	*X*	*X*	932707	0.06696839
	Moraxellaceae	*Streptococcus*	*X*	893147	0.06376892
**cPC4 (gPC8)**	Pasteurellaceae	*Haemophilus*	*influenzae*	3125352	0.68452895
	Staphylococcaceae	*Staphylococcus*	*X*	1060737	−0.54778068
	Neisseriaceae	*X*	*X*	861327	0.32793288
	Moraxellaceae	*X*	*X*	893147	−0.20623383
	Moraxellaceae	*X*	*X*	932707	−0.19733937
	Streptococcaceae	*Streptococcus*	*X*	4479601	−0.11957387
	Corynebacteriaceae	*Corynebacterium*		4474764	−0.09267271
**cPC4 (gPC8)**	Aerococcaceae	*X*	*X*	886735	0.07434957
	Streptococcaceae	*Streptococcus*	*X*	4455633	−0.05788193
	Pasteurellaceae	*Haemophilus*	*influenzae*	2243354	0.05635886

The gene principal components that were identified as differentially-associated with cPCs in the context of RSV severity included gPC1, gPC8, gPC3, gPC13, and gPC12. We identified the top genes from these gPCs based on their loadings (Table 3). Interestingly, we found that 10 of top 20 genes in gPC1 are mitochondrial genes. We also found IFITM1 or IFITM2 in the top loadings of gPC3. These two genes are also significantly differentially-expressed between the acute and post-acute visit, but not between infants with severe versus mild RSV infection (see Fig. 2C). CCR7 (Chemokine receptor type 7), a top gene in gPC1, gPC8, and gPC3, and ILR7 (interleukin 7 receptor) from gPC8 and gPC12 have been found to be downregulated in RSV patients^50^. Other genes present in the loadings of multiple gene PCs included L-ribosomal proteins (RPL family), which are involved in various pathophysiological process, and SELL (L-Selectin), a cell surface lectin mostly expressed in leukocytes, which is down-regulated in RSV infection. SELL also plays a key role in the recruitment of neutrophils to roll along the endothelium to the infected tissue^51,52^.

**Table 3:** Top 20 genes with positive loadings in the gPCs (with associated cPCs in parentheses) identified as associated with significant edges in the sample-specific network analysis.

gPC1 (cPC2)		gPC3 (cPC10)		gPC8 (cPC8 and cPC4)		gPC12 (cPC1)		gPC13 (cPC9)	
Genes	loadings	Genes	loadings	Genes	loadings	Genes	loadings	Genes	loadings
MT-ATP8	0.2631	MT-ATP8	0.4266	CCR7	0.2988	RPL10	0.2631	MT-CO2	0.3230
MT-CO2	0.1894	MALAT1	0.3832	MT-ND4L	0. 2391	TMSB4X	0.1894	B2M	0.2056
ACTB	0.1122	MT-CO2	0.1134	RPS3	0. 2103	MT-ATP8	0.1122	MT-CO3	0.2044
MT-ND4	0.1053	MT-ATP6	0.0846	RPL3	0. 1979	RPL30	0.1053	UCP2	0.1937
MT-CYB	0.0937	MT-CYB	0.0814	RPL10	0. 1977	RPS3	0.0937	RPL10	0.1893
MT-ATP6	0.0865	IFITM2	0.0738	RPL21	0. 1921	TXNIP	0.0865	RPL30	0.1619
CCR7	0.0665	DDX5	0.070	MALAT1	0.1468	RPL14	0.0665	CD52	0.1411
GNB2L1	0.0653	MT-CO1	0.0659	GNB2L1	0.1464	RPS28	0.0653	MT-ND1	0.1297
MT-ND4L	0.0653	MT-ND4	0.0605	MT-CO2	0.1342	CD52	0.0653	RPLP1	0.1181
UCP2	0.0557	IFITM1	0.0543	ARHGDIB	0.1308	RPL19	0.0557	RPS4Y1	0.1076
SELL	0.0521	ACTB	0.0496	UCP2	0.1234	B2M	0.0521	EIF1	0.1014
MT-CO3	0.0513	MT-CO3	0.0482	PABPC1	0.1123	RPL18	0.0513	MALAT1	0.0967
TRAC	0.0506	SELL	0.0312	TRAC	0.1123	IL7R	0.0506	H3F3B	0.0945
RPL3	0.0424	MT-ND5	0.0307	IL7R	0.1017	TRAC	0.0424	RPLP0	0.09342
ARHGDIB	0.0420	MT-ND4L	0.0297	MT-CO3	0.0928	PTMA	0.0420	RPL36	0.0822
MT-CO1	0.0410	MT-ND2	0.0295	RPS28	0.0895	RPS20	0.0410	RPL23A	0.08148
RPL10	0.0397	H3F3B	0.0282	MTND1P23	0.0886	RPL27A	0.0397	GAPDH	0.0814
MT-ND1	0.03553	CCR7	0.0281	HIST1H4C	0.0874	MT-ND6	0.03553	MT-ND4	0.0778
HSPA8	0.0321	IFI44L	0.0281	SNORD13	0.0874	PABPC1	0.0321	RPL18A	0.07362
MT-ND5	0.0301	IFIT3	0.0275	RPS4X	0.0834	TRBV25-1	0.0301	ACTB	0.0721

**Figure 5:**
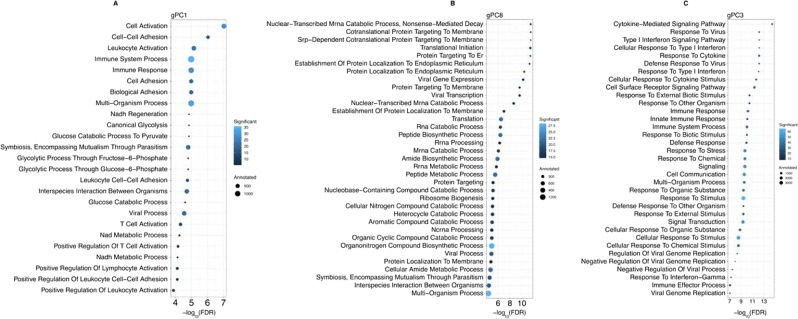
GO term enrichment analysis for the top 100 genes based on the loadings of (**A**) gPC1, (**B**) gPC8, and (**C**) gPC3. These bubble plots include the 25 most significant GO terms identified in each analysis based on the FDR significance. All terms shown are significant at an FDR < 0.05. Size of each bubble indicates the number of genes annotated to the respective GO term and the color indicates the percentage of the top 100 genes annotated to that term. The top 25 GO terms enriched in the other identified gPCs are shown in Supplemental Fig. 6A,B and all GO terms enriched at an FDR < 0.05 are shown in Supplemental Fig. 6C–G.

We performed Gene Ontology (GO) enrichment analysis on the top 100 genes associated with each of these gPCs. For gPC1, top significant terms included “immune system process”, “immune response”, “viral process”, “T cell activation”, and “leukocyte activation”. Terms associated with gPC8 included “viral gene expression”, “viral transcription”, and “multi-organism process”. Terms associated with gPC3 were associated with signaling pathways like “cytokine-mediated signaling”, “type I interferon”, “innate immune response”, “defense response”, “response to other organism”, “viral genome replication”, “defense response”, and “regulation of viral genome replication” (Fig. 5). Similar functions were identified for gPC12 and gPC13 (Supplemental Fig. 6). All of these GO terms are consistent with viral infection and immune response in infected infants. The association of these viral and immune processes with the taxa identified in the associated cPCs allows us to hypothesize that certain microbial species may modulate the immune response in the host CD4^+^ T cells.

## Discussion

Our microbiome data analysis indicated that taxonomic diversity is highest in infants with severe infection during the acute visit, and decreases over time. Moreover, using MaAsLin and at a nominal *p*-value of 0.05, we identified one OTU (*H. influenza*) that is positively associated with RSV severity, and two OTUs (*Ralstonia* and *Streptococcus*) that are negatively associated with RSV severity. These results are partially consistent with previous observations that infants within *H. influenzae*-enriched clusters mount a distinct host inflammatory response characterized by the overexpression of genes related to toll-like receptor signaling and neutrophil recruitment and activation^22^. Interestingly, our subsequent network analysis implicated many of the same OTUs as MaAsLin did. Top OTUs in the network-identified clade PCs included *Streptococcus*, *Corynebacterium*, *Alloiococcus*, *H. influenzae, Staphylococcus*, *Moraxella*, *Ralstonia*, and *Pseudomonas*. These are consistent with a similar study analyzing the microbiome from nasopharyngeal bacterial swabs alongside whole blood transcriptomic data collected from RSV infected infants^22^. In addition, among these identified OTUs, incidence of co-infection of *Streptococcus, Haemophilus influenza,* and *Moraxella* with RSV has been reported in studies of nasopharyngeal aspirate samples from RSV infected infants^53–55^. During acute respiratory illness most infants show stable colonization of these microbes with *Alloiococcus* or *Moraxella*^21^.

Our transcriptome analysis found that genes involved in immune response, such as IFITM1 and IFITM2, decreased in expression after the acute visit, while EZH1, which has also been implicated in immune response signaling^27^, was expressed at lower levels in infants with severe RSV infection. Interferons are widely expressed and are among the key genes responsible for mediating immune response^27,56^. In particular, the IFN-inducible protein is localized in the cell membrane and endocytic vesicle and is important in restricting viral entry to the cell^57–59^. In^27^, IFITM genes have been shown to potentially inhibit RSV infection by interfering with virus entry and subsequent viral multiplication. Despite these promising findings, we note that standard differential expression only identified one gene as significantly (FDR < 0.05) differentially expressed based on RSV infection severity –EZH1 with an FDR of 0.026.

In this paper, we have proposed and applied a novel approach for integrating data from multiple omics platforms, in our case the host transcriptome and nasal microbiome, in order to extract meaningful associations. Our method constructs infant-specific correlation networks between the transcriptome and microbiome in reduced dimensions using PCA. Linear modeling of these interactions, accounting for various confounding factors, allowed us to identify associations between top gene PCs and clade PCs that differ between infants with mild versus severe RSV infection. This allowed us to better understand the etiology of RSV infection and its impact on disease severity by highlighting key associations between active components in the transcriptome and dominant constituents of the microbial composition.

The functional enrichment analysis of the top genes in the network-identified gene PCs indicated that the top-loading genes are highly enriched in pathways related to immune response and viral infection. These top genes included IFITM1 and IFITM2, which were significantly expressed between the acute and post-acute visit, but not between infants with severe versus mild infection, and CCR7, which has been found to be downregulated in RSV patients^50^. Interestingly, many of the top genes are encoded in mitochondrial DNA and included members of NADH dehydrogenase (MT-ND4L and MT-ND5), members of cytochrome c oxidase (MT-CO1, MT-CO2, and MT-CO3), and members of ATP synthase (MT-ATP6 and MT-ATP8). Mitochondrial function can be modulated by viruses^60,61^ and mitochondrial genes play a key role in the host immune response^62,63^. These genes are also important for viral suppression of natural immunity in RSV infection^30^.

Importantly, our analysis indicates that the association of some clade PCs with gene PCs is context-dependent and a function of RSV infection severity. Further, the enrichment of immune and viral pathways in the network-identified gene PCs implies that specific microbial taxa in the nasal microbiome may impact host immune response, potentially mediating RSV infection severity. In this context, the identification of mitochondrial genes in our integrative analysis is an especially intriguing finding. The relationship between mitochondria and the microbiome is only beginning to emerge^64,65^, and has not been previously described in the context of RSV severity. However, interestingly, the role of microbiota has been noted in the immune response to influenza^66^.

We also point out that the sign of the identified associations (negative versus positive correlations) may imply that both adverse as well as cooperative relationships exist between certain bacterial species and immune and viral defense response. For example, clade PC4 and clade PC8 both have positive associations with the gene PC8, indicated a potentially multifactorial host response. However, in the context of this analysis, we note that it is impossible to establish whether the microbiome-transcriptome associations we identified are indeed causal, and, if so, their direction of effect (whether the microbiome influences the transcriptome, vice versa, or both). Without data-collection in infants prior to RSV infection, we can only hypothesize as to whether targeting these associations might help to prevent or minimize the impact of RSV infection.

Finally, it is also important to note that, although highly intriguing, the associations identified in our network analysis were only nominally significant. This likely reflects heterogeneity in the disease as well as a limitation in statistical power due to sample size. However, we point out that the network analysis uncovered very similar microbial taxa as MaAsLin did, lending confidence to our results. Furthermore, highly plausible biological pathways related to viral processes and immune response were implicated in RSV severity using this integrative analysis. These pathways were not found using more standard approaches, such as differential-expression analysis in the context of infection severity, and despite the fact that we did observe some immune genes differentially-expressed between the acute and post-acute visits. We believe these results illustrate the strength of using an integrative analysis approach to bring novel insights into the disease.

This study has several limitations. First, the number of samples with simultaneous collection of transcriptome and microbiome data was small. Hence most of the statistical analysis with interesting results, although nominally significant, did not show significance after adjusted for multiple hypothesis testing. Second, there may have been other host factors such as maternal or household smoking history, breast-feeding status, number of siblings, and antibiotic use, that could affect the severity of RSV infection but were not considered in our study. Third, this study was only able to provide associations between genes and microbiota without exploring the potential causal relationship.

In this study, we performed dimension reduction and differential-association analyses to quantify patterns in the nasal microbiome and host transcriptome that are associated with RSV infection severity. We also used a network-based approach to integrate these two data types and identify higher-order associations among genes and clades. This integrative analysis allowed us to systematically quantify relationships among the nasal microbiome, host transcriptome, and disease severity for RSV infection. Our results suggest that certain associations between the microbiome and transcriptome are modulated based on RSV infection severity, and that particular microbial taxa impact host immune response, with a key role for mitochondrial genes. Overall, these findings on microbiome-transcriptome associations provides novel insights into how the immune system mounts a response against RSV. Based on our findings, a key future direction for our group is to study the potential mechanisms by which the nasal microbiome impacts host immune response, such as how gene regulation is altered or otherwise affected by microbial composition, or how nasal bacteria influence the severity of RSV from a pathophysiologic perspective.

## Methods

### Study participants

RSV-infected infants were enrolled from three cohorts in order to capture the full spectrum of disease severity. A birth cohort was enrolled at the University of Rochester Medical Center’s (URMC), Strong Memorial Hospital and Highland Hospital, and Rochester General Hospital (RGH) for two winter seasons, extending from August 15 to February 1 for 2012–2013 and 2013–2014, and followed by passive and active surveillance for development of RSV infection during the winter months (November 1–April 1). A second cohort was enrolled in pediatric offices or the emergency room at URMC’s Golisano Children’s Hospital or RGH when respiratory symptoms were present. The third cohort was enrolled on admission to the hospital with documented RSV infection. Eligible infants were full-term (>36 weeks gestation), healthy infants born after May 1 and less than 10 months of age at infection.

RSV-infected infants underwent evaluation by two members of the study team (a physician and a nurse). Demographic data, illness symptoms, findings on physical examination, results of laboratory and radiograph results were recorded. A nasal swab was obtained using a medium sized flocked swab (Copan Diagnostics Inc. Murrieta, CA, cat. no. 501CS01) and placed in 2 ml of sterile UV-inactivated water for quantitative RSV reverse-transcriptase polymerase chain reaction (RT-PCR). A 2–3 ml sample of heparinized blood was collected for CD4 gene expression studies as previously described^12^.

### RSV severity score

RSV disease severity was measured using the Global Respiratory Severity Score (GRSS) on a scale from 0–10, with higher scores representing more severe infection^67^. The GRSS was developed using an unbiased data-driven approach with nine clinical variables (including the infant’s general appearance, the presence of wheezing, rales, retractions, cyanosis, lethargy, and poor air movement). The maximal age-adjusted respiratory rate, as well as the worst room air oxygen saturation, are also included in the score. The GRSS is highly predictive of other potential parameters of severity, such as need for hospitalization and duration of hospitalization^67^. The GRSS has an excellent ability to discriminate between mild and severe disease with AUC (area under the receiver operating characteristic curve) ~0.961. An infant with a GRSS ≤ 3.5 (or > 3.5) is classified as having mild (or severe) infection, respectively. For more information see^67^. We note that using continuous or discrete severity scores in our analyses resulted in similar results, so for simplicity we treated RSV infection severity as a binary variable.

### CD4^+^ T cell extraction

CD3^+^, CD4^+^, CD8^−^ T cells were isolated from freshly collected peripheral blood samples as previously described^12,68^. Briefly, within 24 hours of collection, Ficoll-purified peripheral blood mononuclear cells (PBMCs) were stained and sorted into major lymphocyte populations. Sorted cells were immediately lysed and homogenized in RNA extraction buffer and stored for later purification.

### Transcriptomic data collection and analysis

#### RNA extraction, processing and normalization

RNA-Seq data for CD4^+^ T cells were collected at two distinct time points, with 46 samples collected during the acute visit (Visit 1) and 34 samples collected on follow-up day 12–16 (Visit 2). RNA purification, library preparation, sequencing and data processing were essentially the same as previously described^12,68,69^. Briefly, library preparation was performed using the NexteraXT library kit (Illumina, San Diego, CA) following the SMARter Ultra Low amplification kit (Clontech, Mountain View, CA). Libraries were sequenced using the Illumina HiSeq 2500 at a target depth of ~20 million 100-bp single end reads per sample. Raw reads were mapped to Human Genome GRCh38 (annotation of GENCODE 23) and normalized by FPKM (fragments per kilobase of transcript per million reads). The transcriptome data contained reads for 11,576 genes with unique Gene Symbol annotations across 80 total samples.

#### Differential expression analysis

Differential gene expression analysis was performed on FPKM normalized RNA-Seq data using the Bioconductor^70^ *limma* package (version 3.30.13)^71^. We note that principal component (PC) analysis indicated an association of the leading PC with enrollment season. Several methods have been developed to directly regress out signals related to batch in expression data^72–74^. We used ComBat function from sva (version 3.26.0) to correct for differences from enrollment season^75^. In our differential-expression analysis we compared groups based on RSV infection severity (mild vs. severe) while correcting for sex and race. RSV viral load from nasal swab and nasal wash did not show any association with severity of infection^26^ and were not included as covariates. Genes were considered significantly differentially expressed (DE) if their Benjamini-Hochberg corrected p-value was less than 0.05.

#### Differential variability analysis

We used *var.test()* in R (3.4.3 (2017–11–30)) to perform an F-test on log2-transformed gene expression data in order to statistically quantify differences in variance in gene expression levels between the mild and severe RSV-infected groups. Genes were considered significantly differentially variable if their Benjamini-Hochberg corrected p-value was less than 0.05.

#### Gene Ontology pathway analysis

We used the *topGO* package (version 2.30.1)^41^ in R to assess the enrichment of Gene Ontology (GO) pathways in a given gene list. Gene lists analyzed included (1) genes that were identified as significantly differentially-variable (see above), as well as (2) the top 100 genes associated with a gene principal component (PC) from principal component analysis on transcriptome data, based on the associated PC-loadings (see below). In the *topGO (version 2.30.1)* analysis, we used the 11,576 genes with measured reads in our RNA-Seq data as a background.

### Microbiome data collection and analysis

#### 16S rRNA sequencing

Nasal swab specimens were collected during acute RSV-related illness (Visit 1) and one month later (Visit 3). As described in^69^, bacterial 16 S rRNA from these samples was extracted, amplified, and sequenced, and the resulting data were used to determine the taxonomic compositions, in terms of the relative abundances of those present operational taxonomic units (OTUs). Briefly, the V3-V4 hypervariable regions were targeted for amplification and sequenced using an Illumina MiSeq platform according to a paired end 2 × 300 bp read protocol. Preliminary read processing and quality control were performed using the Quantitative Insights into Microbial Ecology (QIIME) software package^76^, and a closed-reference OTU picking was done with USEARCH and the GreenGenes reference database^76^. The final microbiome data contained information for 1,022 distinct OTUs across 91 samples.

#### Multivariate association Analysis with Linear modeling (MaAsLin)

We used MaAsLin (version 0.0.5) to test for significant relationships between microbial clusters and clinical outcome (severe or mild infection). MaAsLin is a multivariate statistical framework that finds associations between clinical metadata and potentially high-dimensional experimental data^76^. In contrast to transcriptomic data, the application of batch correction methods to microbiome data is still nascent^77^. Therefore, when we applied MaAsLin we adjusted for potential confounding factors, including sex and race, as well as enrollment season, which was removed by batch-correction in our transcriptomic data analysis. All microbiome samples from both Visit 1 and Visit 3 were used when running MaAsLin.

### Integrative analysis of transcriptomic and microbiomic data

#### Principal Component Analysis (PCA) on transcriptomic and microbiomic data

To maximize statistical power in the integrative analysis, we first performed dimension reduction^47^. In particular, we applied principal component analysis (PCA) to host transcriptomics data to create a handful of gene principal components (gPCs), and to the nasal microbiomics data to create a few clade principal components (cPCs)^76^. To achieve that, we used the *prcomp()* function in the *stats* (version 3.4.3) package in R to perform PCA on the OTU relative abundance profiles and the FPKM-normalized RNA-Seq gene expression profiles (after applying batch-effect correction for enrollment season). In this integrative analysis, we restricted ourselves to samples from 40 subjects with both microbiome and transcriptome data collected during Visit 1. The top 13 gPCs and top 10 cPCs, which explained 95% of the variance in the transcriptomic and microbiomic data, respectively, were selected for further analysis.

#### Correlation between gPCs and cPCs

We constructed a Spearman correlation matrix comparing the top 13 gPCs and the top 10 cPCs, using *cor()* function from the *stats* (version 3.4.3) package in R. We treated this global 13×10 correlation matrix as the weighted adjacency matrix of a complete bipartite graph (*G*_*α*_, where the subscript *α* denotes the fact that *G*_*α*_ is derived using all the 40 input samples) that contains two types of nodes, gPCs and cPCs.

#### Linear Interpolation to Obtain Network Estimates for Single Samples (LIONESS)

In order to relate gPC/cPC associations to clinical variables, we applied the LIONESS method to *G*_*α*_ to construct sample-specific (infant-specific) correlation networks^49^. LIONESS works under the assumption that the global correlation network represents a linear combination of *N* different networks, one from each of the *N* input samples. Therefore, to construct the network for sample *q*, we first exclude sample *q* and calculate the Spearman correlation matrix using the remaining samples (*G*_(*α*−*q*)_). We then use the LIONESS equation, ${G}_{q}=N({G}_{\alpha }-{G}_{(\alpha -q)})+{G}_{(\alpha -q),}$ to find the network estimate for that sample (*G*_*q*_). We applied LIONESS to the nasal microbiome and host transcriptome samples collected from the same set of 40 infants during Visit 1. The end result of this analysis was 40 sample-specific networks, i.e., bipartite graphs relating gPCs and cPCs.

#### Analysis of sample-specific networks

We separated the sample-specific LIONESS networks based on their severity class and compared the distribution of each edge’s weight between mild and severe groups using *limma* (version 3.34.9) correcting for sex and race. All edges with p-value < 0.05 were considered nominally significant.

## Supplementary information

Supplementary Information

## Data Availability

The Institutional Review Boards of the University of Rochester and Rochester General Hospital approved the study. For each infant, one parent provided written informed consent at enrollment. All methods were performed in accordance with the relevant guidelines and regulations. The data analyzed in the manuscript are available on dbGap (phs001201.v1.p1).
