# Supplementary material for: Microbiome-Transcriptome Interactions Related to Severity of Respiratory Syncytial Virus Infection
Source: Sci Rep. 2019 Sep 25;9:13824. doi: 10.1038/s41598-019-50217-w (PMC6761288; doi:10.1038/s41598-019-50217-w)
Supplement: Supplementary file 1 — Supplementary Information [file 41598_2019_50217_MOESM1_ESM.pdf]

Supplemental Material For:

Microbiome-Transcriptome Interactions Related to Severity of Respiratory Syncytial Virus Infection

Abhijeet R. Sonawane[1]*, Liang Tian[2]*, Chinyi Chu[3], Xing Qiu[4], Lu Wang[4], Jeanne Holden-Wiltse[4], Alex Grier[5], Steven R. Gill[5], Mary T. Caserta[3], Ann R. Falsey[6], David J. Topham[5], Edward E. Walsh[6], Thomas J. Mariani[3], Scott T. Weiss[1], Edwin K. Silverman[1], Kimberly Glass[1#], Yang-Yu Liu[1,7#]

[1]*Channing Division of Network Medicine, Brigham and Women's Hospital and Harvard Medical School, Boston, MA 02115, USA* [2]*Department of Physics, Hong Kong Baptist University, Kowloon Tong, Hong Kong SAR, China* [3]*Departments of Pediatrics,* [4]*Biostatistics and Computational Biology,* [5]*Microbiology and* [6]*Medicine, University of Rochester Medical Center, Rochester, NY 14642, USA* [7]*Center for Cancer Systems Biology, Dana Farber Cancer Institute, Boston, MA 02115, USA*

*There authors contributed equally to this work.
[#]Correspondence should be addressed to KG (kimberly.glass@channing.harvard.edu) or YYL (yyl@channing.harvard.edu)

Supplemental Figure Legends

Supplemental Figure 1: Analysis of comparison of viral load data and hospitalization against disease severity. We plot the RSV severity score GRSS against viral load values for the samples collected from nasal swab and nasal wash. We did not find any evidence for disease severity being affected by viral load. The points were colored according to hospitalization status (red= hospitalized and black = Not hospitalized). The dashed line represent classification of severity in mild and severe groups based on GRSS.

Supplemental Figure 2: PCA and MDS (multidimensional scaling) on transcriptome data. Principal component analysis was performed on all 80 transcriptomic samples using the log2 normalized RNA-Seq FPKM data and MDS plots for the same samples were obtained using the plotMDS() function in *limma*. For each analysis, points in the plots have been colored to indicate the corresponding sample's severity, sex, enrollment season, race, ethnicity, and visit number. The left hand panel shows results for PCA and MDS performed on the transcriptomic data prior to batch-correction; the right hand panel shows the analyses after batch-correcting the transcriptomic data for enrollment season.

Supplemental Figure 3: Gene enrichment results for 641 genes with significant (FDR<0.05) differentially variable expression levels between mild and severe samples, as measured using the F-test.

Supplemental Figure 4: PCoA on microbiome data. Scatterplots from principal coordinate analysis (PCoA), based on Euclidean, rJSD, unweighted Unifrac, weighted Unifrac distances of the OTUs in nasal microbiome samples from infants with RSV infection are shown and points colored to indicate the corresponding sample's severity, sex, enrollment season, race, ethnicity, and visit number.

Supplemental Figure 5: Sensitivity analysis on single-sample network edges. We plot the percentage of times an edge is identified as significant out of 1000 iterations of *limma* analysis comparing random subsets with 10 mild and 10 severe samples, corrected for sex and race. Color corresponds to direction of fold change for significant edges. The line at 5% indicates the percent occurrence expected by chance.

Supplemental Figure 6: GO term enrichment analysis for the top 100 genes based on the loadings of (**A**) gPC13, (**B**) gPC12 and (**C**) gPC8. The top 25 most significant GO terms, based on their FDR, are shown (all terms meet a significance level of FDR<0.05) (**D-H**) Plots showing all significant GO terms (FDR<0.05) identified for (**D**) gPC1, (**E**) gPC8, (**F**) gPC3, (**G**) gPC13, and (**H**) gPC12. Size of each bubble indicates the number of genes annotated to the respective GO term and the color indicates the percentage of the top 100 genes annotated to that term.

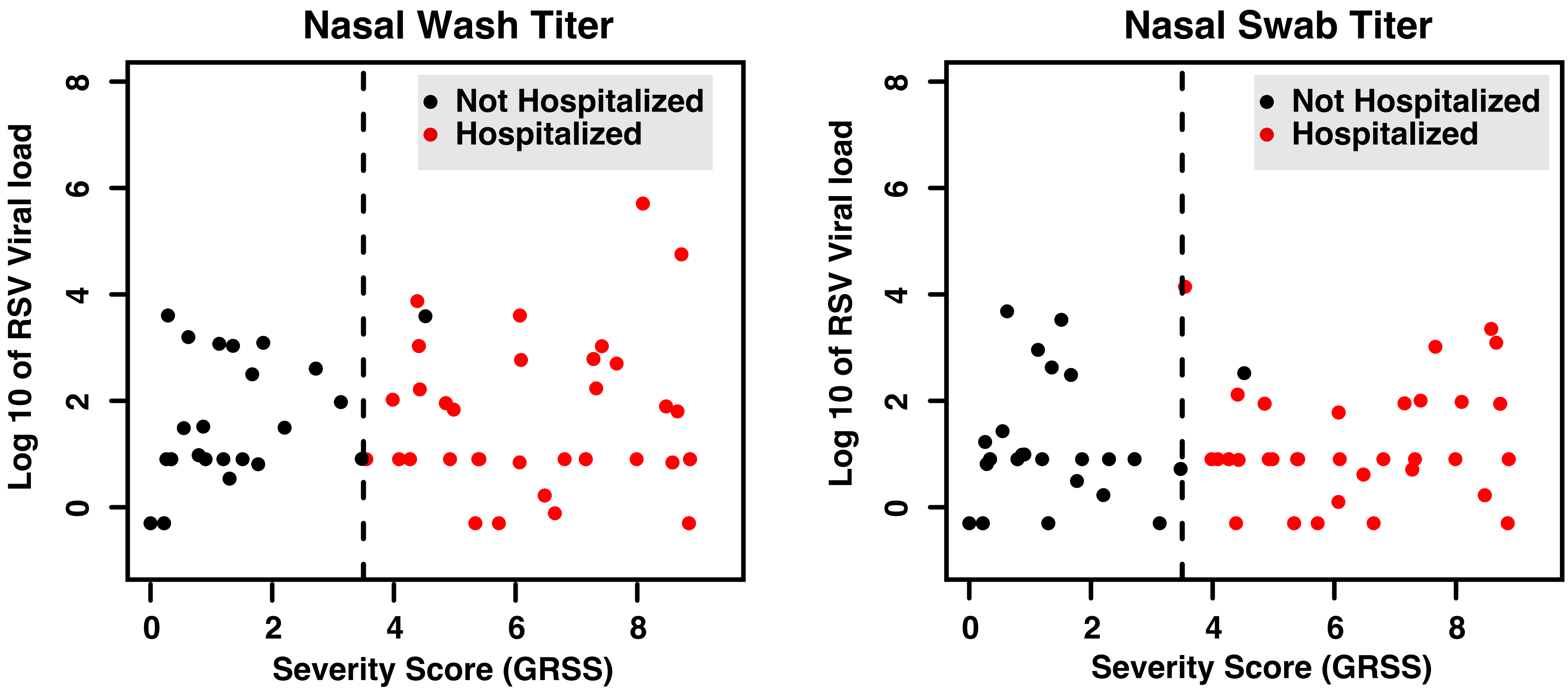
Nasal Wash Titer
Not Hospitalized
Hospitalized
Log 10 of RSV Viral load
Severity Score (GRSS)
0
2
4
6
8
Nasal Swab Titer
Not Hospitalized
Hospitalized
Log 10 of RSV Viral load
Severity Score (GRSS)

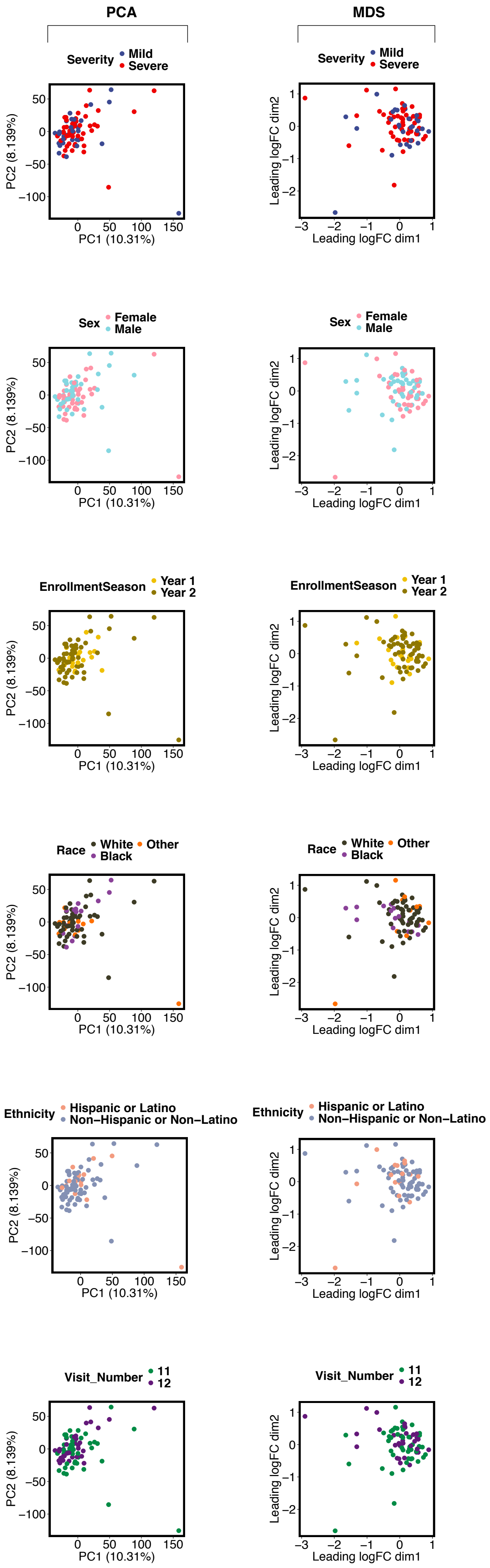
PCA
MDS
Severity
Mild
Severe
Sex
Female
Male
EnrollmentSeason
Year 1
Year 2
Race
White
Other
Black
Ethnicity
Hispanic or Latino
Non−Hispanic or Non−Latino
Visit_Number
11
12
PC2 (8.139%)
PC1 (10.31%)
Leading logFC dim2
Leading logFC dim1

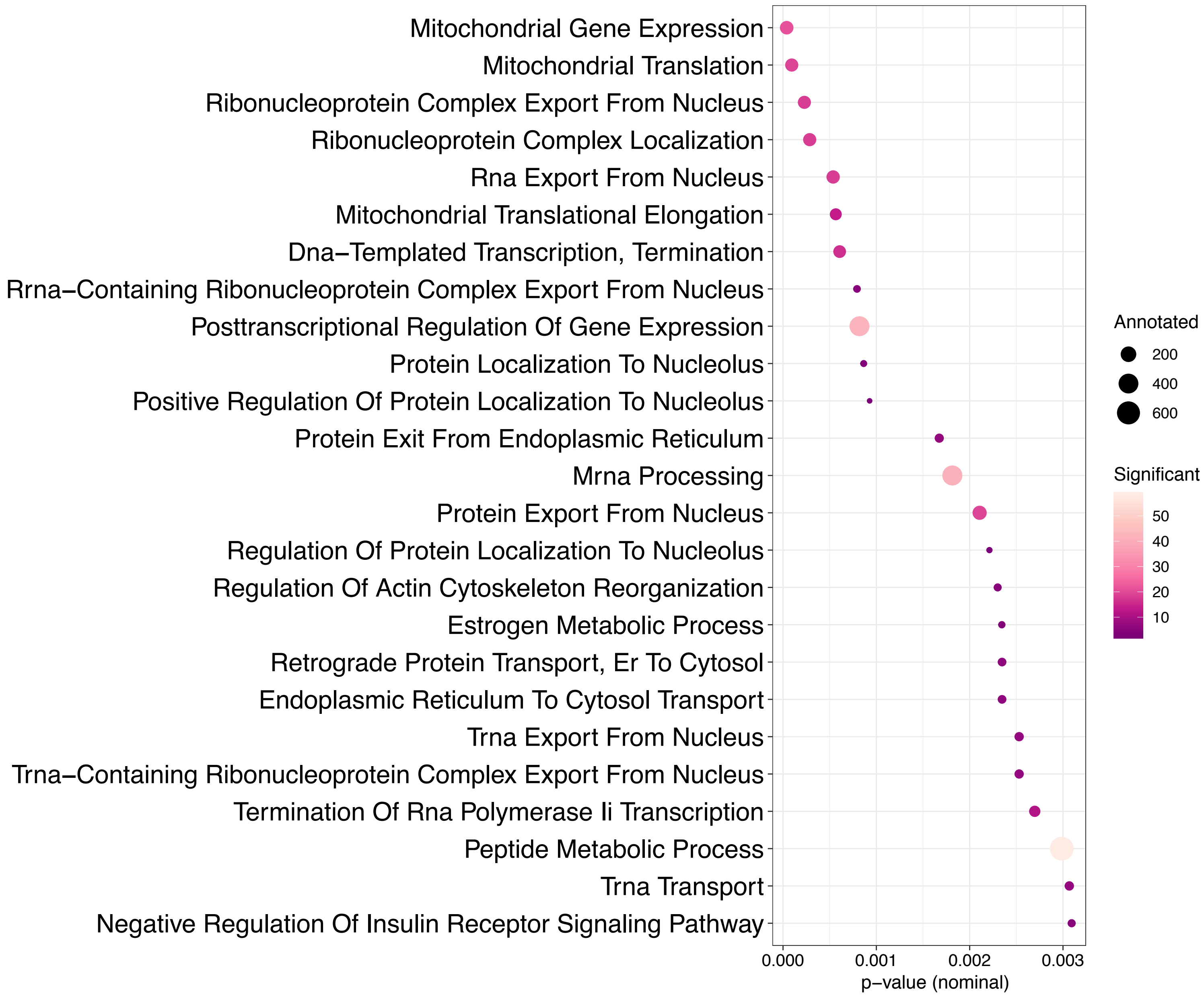
Mitochondrial Gene Expression
Mitochondrial Translation
Ribonucleoprotein Complex Export From Nucleus
Ribonucleoprotein Complex Localization
Rna Export From Nucleus
Mitochondrial Translational Elongation
Dna–Templated Transcription, Termination
Rrna–Containing Ribonucleoprotein Complex Export From Nucleus
Posttranscriptional Regulation Of Gene Expression
Protein Localization To Nucleolus
Positive Regulation Of Protein Localization To Nucleolus
Protein Exit From Endoplasmic Reticulum
Mrna Processing
Protein Export From Nucleus
Regulation Of Protein Localization To Nucleolus
Regulation Of Actin Cytoskeleton Reorganization
Estrogen Metabolic Process
Retrograde Protein Transport, Er To Cytosol
Endoplasmic Reticulum To Cytosol Transport
Trna Export From Nucleus
Trna–Containing Ribonucleoprotein Complex Export From Nucleus
Termination Of Rna Polymerase Ii Transcription
Peptide Metabolic Process
Trna Transport
Negative Regulation Of Insulin Receptor Signaling Pathway
0.000
0.001
0.002
0.003
p–value (nominal)
Annotated
200
400
600
Significant
50
40
30
20
10

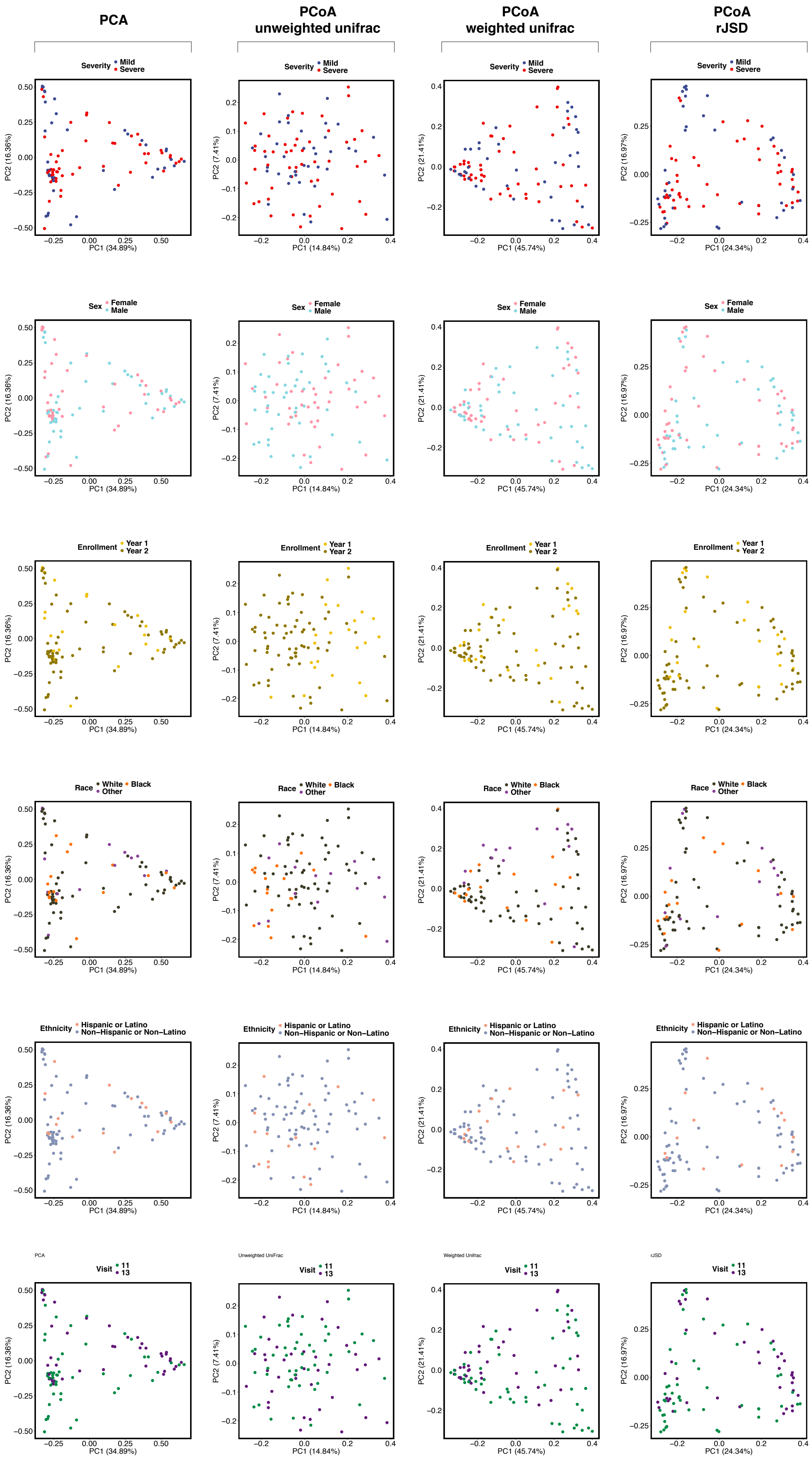

PCA
PCoA
unweighted unifrac
PCoA
weighted unifrac
PCoA
rJSD
Severity
Mild
Severe
Sex
Female
Male
Enrollment
Year 1
Year 2
Race
White
Black
Other
Ethnicity
Hispanic or Latino
Non−Hispanic or Non−Latino
PCA
Unweighted UniFrac
Weighted Unifrac
rJSD
Visit
11
13
PC1 (34.89%)
PC2 (16.36%)
PC1 (14.84%)
PC2 (7.41%)
PC1 (45.74%)
PC2 (21.41%)
PC1 (24.34%)
PC2 (16.97%)

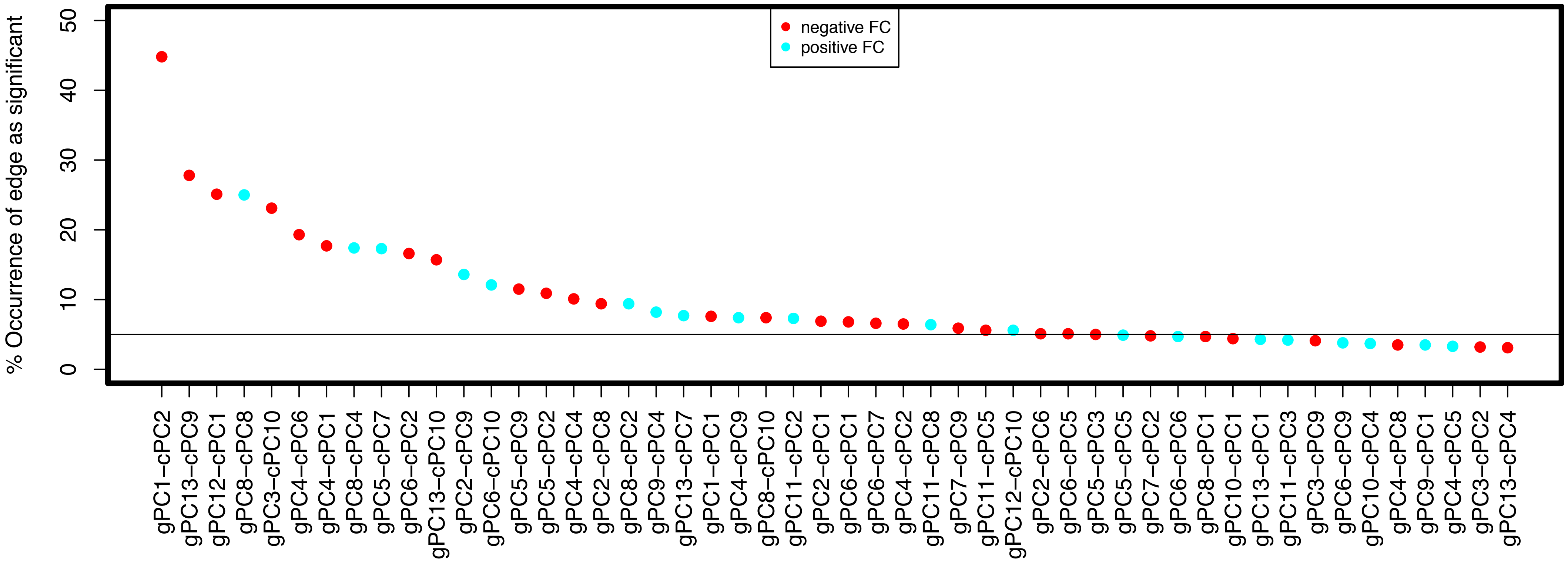

% Occurrence of edge as significant
0
10
20
30
40
50
negative FC
positive FC
gPC1−cPC2
gPC13−cPC9
gPC12−cPC1
gPC8−cPC8
gPC3−cPC10
gPC4−cPC6
gPC4−cPC1
gPC8−cPC4
gPC5−cPC7
gPC6−cPC2
gPC13−cPC10
gPC2−cPC9
gPC6−cPC10
gPC5−cPC9
gPC5−cPC2
gPC4−cPC4
gPC2−cPC8
gPC8−cPC2
gPC9−cPC4
gPC13−cPC7
gPC1−cPC1
gPC4−cPC9
gPC8−cPC10
gPC11−cPC2
gPC2−cPC1
gPC6−cPC1
gPC6−cPC7
gPC4−cPC2
gPC11−cPC8
gPC7−cPC9
gPC11−cPC5
gPC12−cPC10
gPC2−cPC6
gPC6−cPC5
gPC5−cPC3
gPC5−cPC5
gPC7−cPC2
gPC6−cPC6
gPC8−cPC1
gPC10−cPC1
gPC13−cPC1
gPC11−cPC3
gPC3−cPC9
gPC6−cPC9
gPC10−cPC4
gPC4−cPC8
gPC9−cPC1
gPC4−cPC5
gPC3−cPC2
gPC13−cPC4

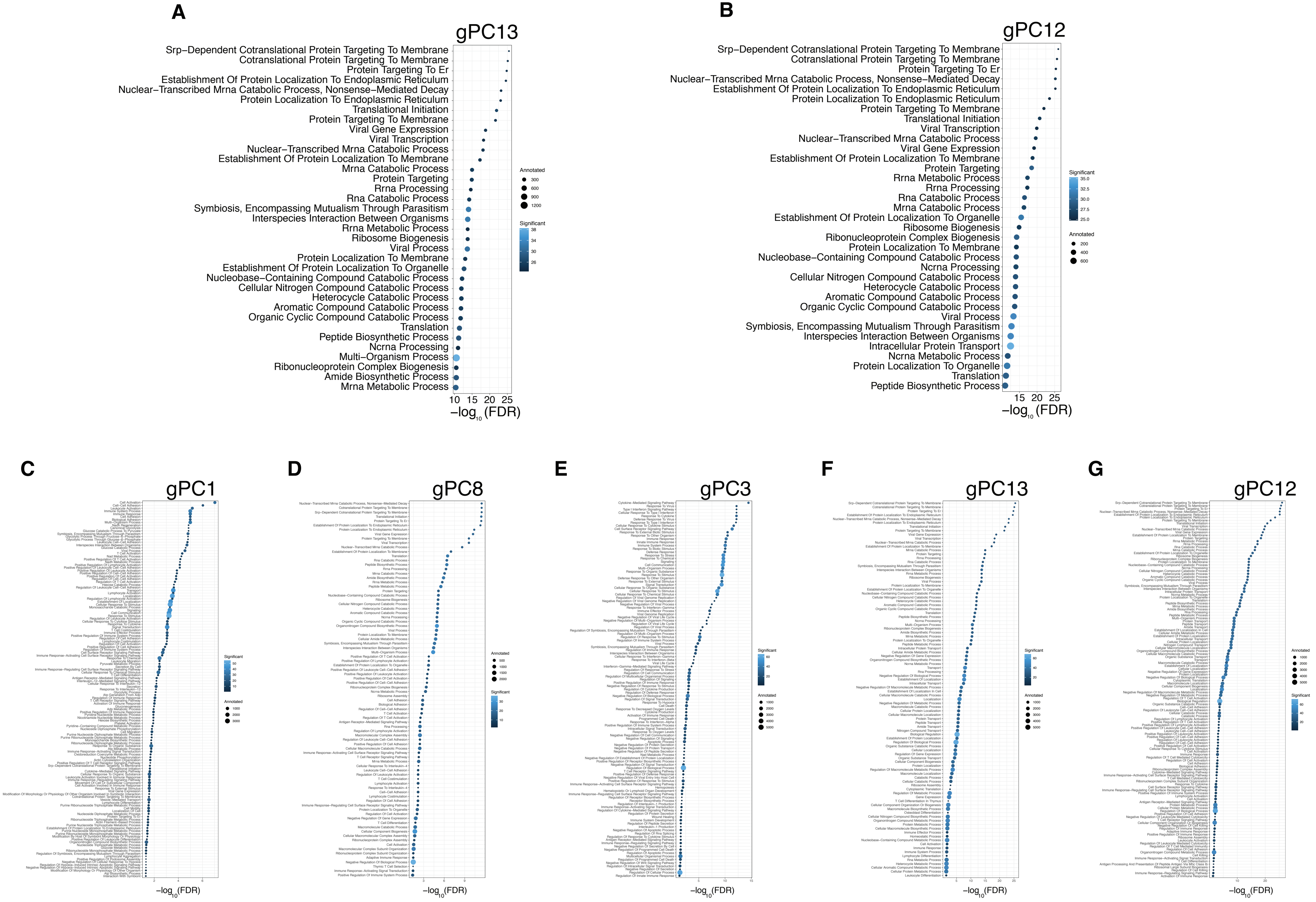
A
gPC13
Srp−Dependent Cotranslational Protein Targeting To Membrane
Cotranslational Protein Targeting To Membrane
Protein Targeting To Er
Establishment Of Protein Localization To Endoplasmic Reticulum
Nuclear−Transcribed Mrna Catabolic Process, Nonsense−Mediated Decay
Protein Localization To Endoplasmic Reticulum
Translational Initiation
Protein Targeting To Membrane
Viral Gene Expression
Viral Transcription
Nuclear−Transcribed Mrna Catabolic Process
Establishment Of Protein Localization To Membrane
Mrna Catabolic Process
Protein Targeting
Rrna Processing
Rna Catabolic Process
Symbiosis, Encompassing Mutualism Through Parasitism
Interspecies Interaction Between Organisms
Rrna Metabolic Process
Ribosome Biogenesis
Viral Process
Protein Localization To Membrane
Establishment Of Protein Localization To Organelle
Nucleobase−Containing Compound Catabolic Process
Cellular Nitrogen Compound Catabolic Process
Heterocycle Catabolic Process
Aromatic Compound Catabolic Process
Organic Cyclic Compound Catabolic Process
Translation
Peptide Biosynthetic Process
Ncrna Processing
Multi−Organism Process
Ribonucleoprotein Complex Biogenesis
Amide Biosynthetic Process
Mrna Metabolic Process
10 15 20 25
−log10 (FDR)
Annotated
300
600
900
1200
Significant
38
34
30
26
B
gPC12
Srp−Dependent Cotranslational Protein Targeting To Membrane
Cotranslational Protein Targeting To Membrane
Protein Targeting To Er
Nuclear−Transcribed Mrna Catabolic Process, Nonsense−Mediated Decay
Establishment Of Protein Localization To Endoplasmic Reticulum
Protein Localization To Endoplasmic Reticulum
Protein Targeting To Membrane
Translational Initiation
Viral Transcription
Nuclear−Transcribed Mrna Catabolic Process
Viral Gene Expression
Establishment Of Protein Localization To Membrane
Protein Targeting
Rrna Metabolic Process
Rrna Processing
Rna Catabolic Process
Mrna Catabolic Process
Establishment Of Protein Localization To Organelle
Ribosome Biogenesis
Ribonucleoprotein Complex Biogenesis
Protein Localization To Membrane
Nucleobase−Containing Compound Catabolic Process
Ncrna Processing
Cellular Nitrogen Compound Catabolic Process
Heterocycle Catabolic Process
Aromatic Compound Catabolic Process
Organic Cyclic Compound Catabolic Process
Viral Process
Symbiosis, Encompassing Mutualism Through Parasitism
Interspecies Interaction Between Organisms
Intracellular Protein Transport
Ncrna Metabolic Process
Protein Localization To Organelle
Translation
Peptide Biosynthetic Process
15 20 25
−log10 (FDR)
Significant
35.0
32.5
30.0
27.5
25.0
Annotated
200
400
600
C
gPC1
−log10 (FDR)
D
gPC8
−log10 (FDR)
E
gPC3
−log10 (FDR)
F
gPC13
−log10 (FDR)
G
gPC12
−log10 (FDR)
